# Impact of Treadmill Interval Running on the Appearance of Zinc Finger Protein FHL2 in Bone Marrow Cells in a Rat Model: A Pilot Study

**DOI:** 10.3390/life12040528

**Published:** 2022-04-02

**Authors:** Alexandre Germain, Celine Bourzac, Chantal Pichon, Hugues Portier, Stéphane Pallu, Philippe Germain

**Affiliations:** 1 Centre de Biophysique Moléculaire (CBM), UPR CNRS 4301, Université d’Orléans, Rue Charles Sandron, CS 80054, F-45071 Orléans, France; alexandre.germain@univ-orleans.fr (A.G.); chantal.pichon@cnrs.fr (C.P.); 2 UFR Science & Technique, Université d’Orléans, 2 Allée du Château, F-45100 Orléans, France; hugues.portier@univ-orleans.fr (H.P.); stephane.pallu@univ-orleans.fr (S.P.); 3 Laboratoire de Biologie, Bioingénierie et Bioimagerie Ostéo-Articulaires (B3OA), UMR CNRS 7052, INSERM U1271, Université de Paris, 10 Avenue de Verdun, F-75010 Paris, France; celine.bourzac@vet-alfort.fr; 4 Plateforme de Recherche Biomédicale, Ecole Nationale Vétérinaire d’Alfort, 7 Avenue du Général de Gaulle, F-94700 Maisons-Alfort, France

**Keywords:** treadmill running, bonne marrow, FHL2, signaling pathway, mechano-transduction, rat

## Abstract

Although the benefits of physical exercise to preserve bone quality are now widely recognized, the intimate mechanisms leading to the underlying cell responses still require further investigations. Interval training running, for instance, appears as a generator of impacts on the skeleton, and particularly on the progenitor cells located in the bone marrow. Therefore, if this kind of stimulus initiates bone cell proliferation and differentiation, the activation of a devoted signaling pathway by mechano-transduction seems likely. This study aimed at investigating the effects of an interval running program on the appearance of the zinc finger protein FHL2 in bone cells and their anatomical location. Twelve 5-week-old male Wistar rats were randomly allocated to one of the following groups (*n* = 6 per group): sedentary control (SED) or high-intensity interval running (EX, 8 consecutive weeks). FHL2 identification in bone cells was performed by immuno-histochemistry on serial sections of radii. We hypothesized that impacts generated by running could activate, in vivo, a specific signaling pathway, through an integrin-mediated mechano-transductive process, leading to the synthesis of FHL2 in bone marrow cells. Our data demonstrated the systematic appearance of FHL2 (% labeled cells: 7.5%, *p* < 0.001) in bone marrow obtained from EX rats, whereas no FHL2 was revealed in SED rats. These results suggest that the mechanical impacts generated during high-intensity interval running activate a signaling pathway involving nuclear FHL2, such as that also observed with dexamethasone administration. Consequently, interval running could be proposed as a non-pharmacological strategy to contribute to bone marrow cell osteogenic differentiation.

## 1. Introduction

In the fight against degeneration of the musculoskeletal system, non-drug prophylactic approaches, particularly through physical activity, hold a very promising place [1], and the connection between them and good health or wellness is now admitted as a part of an integrative approach [2]. The intimate mechanisms by which physical exercise induces structural musculo-skeletal adaptations, however, need to be further investigated [3], and specifically the place of the mechano-transductive signalling pathways induced by mechanical stress of the musculo-skeletal system need to be better defined [4]. Indeed, the mechanical stresses applied to the cells induce changes in their structure and shape by altering the balance of strains at the cell level, and these strains generate various cell responses, such as growth, differentiation, mobility, remodelling, and gene expression for instance, thus determining the cell fate [5,6,7]. Cells implement the mechano-transductive process by converting perceived physical stimuli into intracellular biochemical signals [8,9]. These biochemical signals may consist of the activation of a specific signalling transduction pathway, which results in the adaptation of the cell to the physical stimuli [10].

Regarding bone specifically, physical exercise is usually known to have an anabolic effect on bone tissue, mineral density (BMD), or global bone status [11]. Different exercises have, however, different effects on bone status. In particular, the best osteogenic effects have been observed in sports in which bone strains resulted from impacts [12]. Outdoor running [13] or treadmill running [14] are consequently good candidates to induce bone adaptations.

To explain the relationship between physical activities and mechano-transductive processes, various prospective actors have been investigated, including the pressure generated by the interstitial fluid bathing the osteocytes and its effects on osteoblast and osteoclast activities [15], or the role played by bone marrow cells [16]. Indeed, mesenchymal stem cells (MSCs) are multipotent stromal cells capable of multilineage differentiation. They contribute to the regeneration or the repair of mesenchymal tissues such as bone, cartilage, muscle, ligament, tendon, and adipose tissue [17]. Various signaling transduction pathways driving the differentiation of bone marrow MSCs toward the osteogenic lineage are described [18].

Several proteins involved in the mechano-transduction processes affecting MSCs, fibroblasts, chondrocytes, osteocytes, and myoblasts, have been studied [19,20,21,22,23]. It appears that the regulatory effects of an extracellular matrix (ECM) mechanical stress on cell signalling pathways could be mediated by integrins, Focal Adhesion Kinase (FAK), G proteins, receptor tyrosine kinases, mitogen-activated protein kinases (MAPK), and stretch-activated ion channels [24]. Concerning integrins, using peptide RGD (sequence (Arg-Gly-Asp)), a continuum from the ECM (i.e., type I collagen, fibronectin, laminin, thrombospondin and vitronectin) to the intracellular cytoskeleton, has been modelized in human bone marrow MSCs to increase cell adhesion on biomaterials [25].

More recently, it has been shown that zinc finger proteins, and especially their LIM domain [26], could be involved in a mechano-sensing response [27] and could induce gene-transcription regulation [28]. They would therefore be reasonable candidates to participate in a mechano-transductive process.

Four and a half LIM protein type 2 (FHL2) belong to this class of proteins. FHL2 is detected closed to Actin filaments and Focal adhesion of the cytoskeletal subcellular compartment. In reference to the “Human Protein Atlas” [29], FHL2 is expressed in small amounts in hematopoietic cells of bone marrow. It is a multifunctional intracellular adaptor protein expressed in cells, which participates in various cell processes [30] including adjustments in signalling cascades and gene transcription activities [31]. This protein is involved in muscle development [32], cardiovascular system hypertrophy, atherosclerosis or angiogenesis [33], changes in chondrocyte morphology [34], mesenchymal cell osteogenic differentiation and bone formation [35], fibroblast activation [36], or adipocyte differentiation [37]. In vitro, in the absence of mechanical stresses, FHL2 appears in bone tissue in response to chemical osteoinductive stimulation (dexamethasone [38]), which mediates MSC differentiation into osteoprogenitor cells. FHL2 expression would therefore induce Alkaline phosphatase, type I collagen and osteocalcin synthesis, as well as extracellular matrix mineralization [39]. It is suggested by Nakazawa et al., 2016, [40] that FHL2 phosphorylation by FAK is a critically dependent mechanical step in signalling from the extracellular matrix to the nucleus for gene expression and cell proliferation. Moreover, if FHL2 is important for bone marrow MSC differentiation, it must be underlined that it is also essential to the maintenance of bone Hematopoietic Stem Cells (HSCs) in a quiescent state and to their survival under biochemical stress conditions [41].

Krüppel-like factor 8 (KLF8) is a zinc finger protein identified as a transcriptional factor [42]. It is also a target of the FAK signalling pathway for up-regulation of the cyclin D1 promotor [42]. This protein is localized in the nucleus of bone marrow cells in reference to the Human Protein Atlas [29], but also in the cytosol and nucleoplasm for the UniProt Database [43]. It plays a crucial role in differentiation or proliferation of several kinds of cells [44,45].

Thus, FAK plays a major role in mediating signal transduction by integrin [46] with the consequence of transmitting signals from the extracellular matrix to the cytoskeleton [25,44] and to the nucleus. Moreover, FAK has several interactions with KLF8 or FHL2 activities.

Consequently, in reference to the in vitro signalling pathways listed by Langenbach and Handschel. (2013) [47], which induce the osteogenic differentiation of stem cells by increasing the transcription of FHL2, we hypothesized that mechanical stress impacts generated by interval running could activate, in vivo, a FAK-dependent signaling pathway. This mechanism could be expected to pass by an integrin-mediated mechano-transductive process, leading to KLF8 up-regulation or activation and FHL2 synthesis or translocation with consequences for bone marrow cell osteogenic differentiation [16].

## 2. Materials and Methods

### 2.1. Animal Experiment

Twelve 5-week-old male Wistar rats, weighing 203 ± 10 grams, were purchased from Elevage Janvier (Le Genet-St-Isle, France), acclimated for 1 week to the new facilities and for 1 week to the treadmill. Rats were then randomly assigned to one of the 2 following groups (*n* = 6 each): sedentary control (SED) or running exercise (EX).

At the beginning of the experiment, all rats were housed in controlled facilities (3 per standard cage) and maintained on a 12 h light/dark cycle, at a constant temperature of 21 ± 2 °C. A commercial standard diet (Genestil, Royaucourt, France) and tap water were provided *ad libitum* to all animals.

The experimental study was carried out in strict accordance with the European Guidelines for Care and Use of Laboratory Animals (Directive 2010/63/EU). The experimental protocol received approval from the Ethics Committee on Animal Research of Lariboisière/Villemin (Paris, France) and the French Ministry of Agriculture (Paris, France; APAFIS # 9505).

### 2.2. Maximal Aerobic Speed Test

On the 5th day of acclimation, each rat underwent Maximal Aerobic speed (MAS) testing, using a progressive running test [48] to determine subsequent running speeds. It started with a warming session at 10° inclination and a speed of 13 m/min for 5 min, followed by increments of 4 m/min every 2 min until 17 min were reached, then increments of 4 m/min every 1 min 30 s. The test was conducted until the rats could no longer keep pace with the treadmill speed, despite 2 consecutive stimulations with air-compressed sprays. The test was therefore stopped, and the last fully sustained increment speed defined as the MAS for the rat.

The same MAS test was conducted at the end of the exercise protocols to validate the exercise program.

### 2.3. Training Protocol

The running training protocol was performed on the treadmill: 0° inclination, 45 min per day, 5 days, for 8 consecutive weeks. It consisted of 7 repetitions in blocks of 3 min at a moderate speed (70% MAS), followed by 2 min of high-intensity running (i.e., 100% MAS) and 1 min of passive recovery.

### 2.4. Bone Histology

One day after the last training session, the animals were euthanized by exsanguination and then the radii were excised, cleared of connective soft tissues, and fixed in 4% *v*/*v* paraformaldehyde at 4 °C. Bone samples were slowly decalcified with EDTA 177 g/L, pH 7.0–7.3 (Osteosoft, Merck KGaA, Darmstadt, Germany), embedded in paraffin, and cut longitudinally with a microtome.

Slides were mounted, and immunohistochemical labelling was performed with a primary anti-FHL2 antibody (EPR17860-23, abcam) for the whole different bone tissues, or with anti-KLF8 antibody (PAS-67196, Invitrogen) for the bone marrow, at the dilution 1/100 for both antibodies. 3,3′-Diaminobenzidine (3,3′-DAB) was added for stain revelation and counter-staining was achieved with hematoxylin.

### 2.5. Imaging Processing

Slides were observed under an optical microscope with a camera at 20× magnification and images were visualized on a computer screen using IC Capture^®®^ software (The imaging Source Europe GmbH, Sommerstrasse, Germany) for image acquisition (v.2.4, exposure time 1/6410 s; 8.81 db gain; acuity 0; gamma 48).

Ten pictures for cortical, trabecular, and bone marrow compartments, respectively, were defined for each rat.

**Figure 1:**
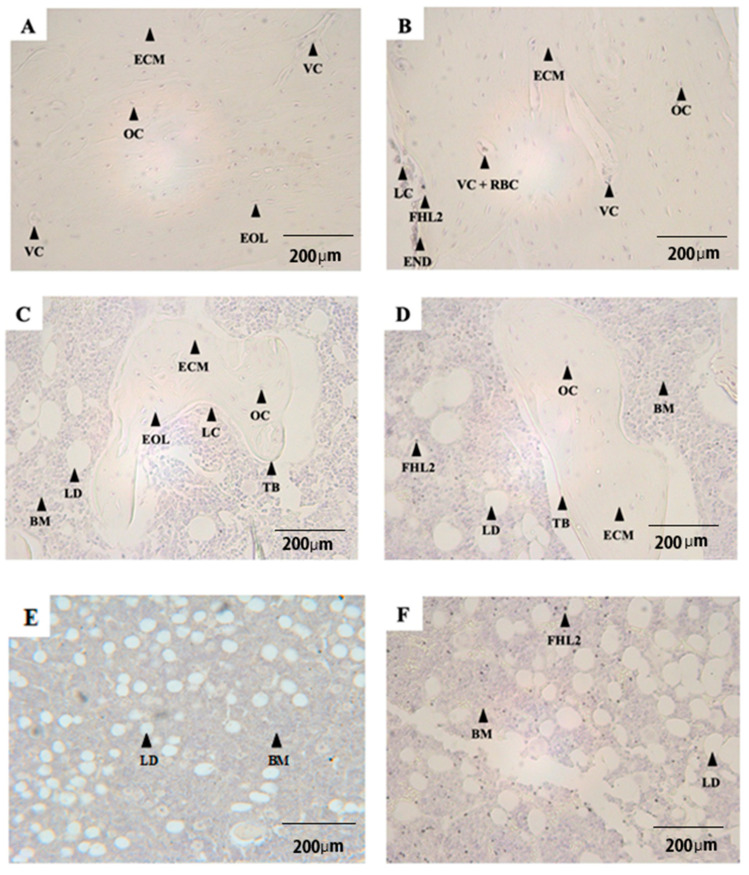

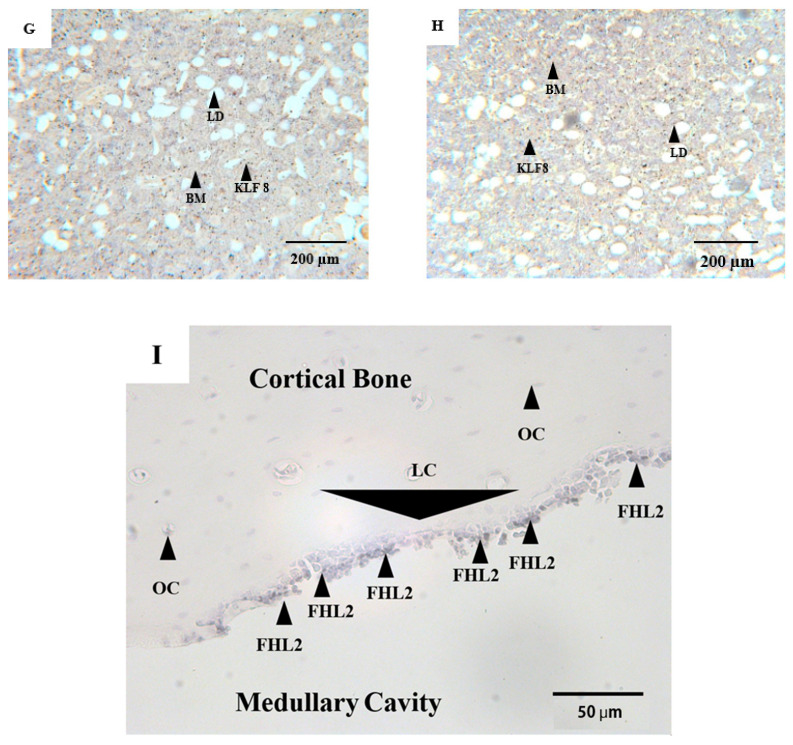
Longitudinal serial sections under light microscopy at 200× magnification (3,3′-Diaminobenzidine stained and hematoxylin counterstained): cortical parts of radii from SED (**A**) and EX (**B**) rats; trabecular parts and bone marrow of radii from SED (**C**) and EX (**D**) rats; bone marrow of radii from SED (**E**) and EX (**F**) rats. Each arrowhead points at a specific structure: OC, osteocyte; VC, vascular canal; ECM, extracellular matrix; EOL, empty osteocyte lacuna; TB, trabecular bone; RBC, red blood cells; LC, lining cells; END, endosteum; BM, bone marrow; LD, lipid droplet; FHL2, four and half LIM protein type 2. Longitudinal serial sections of the bone marrow under light microscopy at 100× magnification (3,3′-Diaminobenzidine stained and hematoxylin counterstained): bone marrow of radii from SED (**G**) and EX (**H**) rats. Each arrowhead points at a specific structure: BM, bone marrow; LD, lipid droplet; KLF8, Krüppel-like factor 8 protein. Longitudinal serial sections under light microscopy at 200× magnification (3,3′-Diaminobenzidine stained and hematoxylin counterstained): Cortical Bone of radii from EX (**I**) rats. Each arrowhead points at a specific structure: CB; Cortical Bone; FHL2, four and half LIM protein type 2; LC, Lining cells; OC, Osteocyte.

Qualitative analyses were performed for bone architecture and bone marrow composition, as reported by Lapidot et al., 2005 [49]. (Different parameters are shown in the Figure 1 osteocyte; vascular canal; extracellular matrix; empty osteocyte lacuna; trabecular bone; red blood cells; lining cells; endosteum; bone marrow; lipid droplet, FHL2, KLF8.)

For quantitative analyses, pictures from the bone sections were digitally processed and the total number of cells were counted in each picture field using Image J software (National Institute of Health, Bethesda, MD, USA, 1987) and associated digital processing plug-in. Image processing was performed as follows: The background noise of the image was first subtracted. The image was converted into 16-bit images, and then into binary images with a contrast adjusted to 27% of the total signal. The binary processing was set up with the “Files Holes” mode, which allowed the removal of signals of a too-low intensity (empty areas). Finally, the number of structures exceeding 150 pixels and delimited with the “analyse particles” mode were counted, excluding the structures too near to the edges of the image. The number of marked cells (i.e., labelled by immuno-histochemistry) and the number of osteocytes (easily identified in their lacunae) were counted manually on printed pictures.

### 2.6. Statistical Analysis

We tested that our data followed a normal distribution (Shapiro–Wilk test) and then we verified for each condition that the data distribution followed this law.

Moreover, we also completed a Fisher–F test to control variance homogeneity.

The comparison between groups was performed using a quantile–quantile diagram or “Q-Q plot”.

To complete this first overview of the normality of our data, we submitted our data to the Shapiro–Wilk test (“Statistical Tools For High-Throughput Data Analysis” platform: sthda.com; accessed on 15 Jun 2019). We used as a null hypothesis the follow-up of a normal distribution. Our confidence interval was set at 95%.

We then performed a comparison of the mean percentages of cells labelled with each primary antibody. Since our samples came from different animals, our data were distributed according to a normal distribution; there was homogeneity of the variances, and we opted for a parametric Student’s “*t*” test for independent groups, on a bilateral basis (tests performed on the biostatgv.sentiweb.fr platform). Our null hypothesis was that the means were equal in both groups and our confidence interval was set at 95%. Variables were expressed as means ± SEM.

## 3. Results

### 3.1. Qualitative Observations

Pictures A and B in Figure 1 display cortical bone from both SED and EX groups. Osteocytes appeared purple in their lacunae. Compared to the SED group (A), a low number of empty osteocyte lacunae were observed in the EX group (B). In contrast, no differences in the number and size of vascular canals were observed among groups. These parameters served to locate FHL2 or KLF8 immuno-staining in different experimental conditions.

A slight visual difference in FHL2 immunostaining between the SED and EX groups was observed.

Figure 1C,D display trabecular bone in the diaphyseal medullary canal, embedded in the marrow, including MSC and HSC (which cannot be distinguished in the pictures).

Osteocytes in the trabecular bone were stained in purple by hematoxylin. Although particularly rare, in the cortical bone, FHL2 labelling was observed.

Figure 1E,F display bone marrow in the medullar canal.

In the EX group, FHL2 immunostaining intensity in bone marrow appears stronger than respective results obtained in cortical bone (Figure 1B versus Figure 1F). Moreover, this labelling was not randomly distributed, but rather located in the diaphysis (Figure 1E,F), whereas rare labelling was observed (for example, in the epiphyses). The DAB labelling combined with the FHL2 antibody and counter-staining with hematoxylin looks specific to the same subcellular compartment (the cell nucleus).

Compared to the SED group, a stronger labelling intensity of FHL2 was observed in the EX group (Figure 1F), whereas no difference in KLF8 immunostaining appeared between SED and EX groups in the bone marrow (Figure 1G,H).

Furthermore, the labelling in the lining cells (Figure 1I) in the endosteal region was only observed in the diaphyseal part, but not in the metaphyseal or epiphyseal cortical parts. No labelling was observed in the osteocytes. It seemed, however, that the labelling could be observed in blood cells in the vascular canal.

### 3.2. Quantitative Analyses

In cortical bone, the percentage of FHL2-labelled cells in the EX group (1.3 ± 1.2%) was higher (*p* < 0.05) than the respective results obtained in the SED group where no FHL2 was stained.

In the trabecular bone, the percentage of FHL2-labelled cells was 0.2 ± 0.08% and 0% in the EX and SED groups, respectively. The difference was, however, not statistically significant.

In the bone marrow, the percentage of FHL2 labelled cells in the EX group was significantly higher (*p* < 0.05) than those obtained in the SED group (7.5 ± 1.6% vs. 0%).

In the bone marrow, the percentage of KLF8 labelled cells in the EX group was not significantly higher than results obtained in the SED group (14 ± 4.2% vs. 12.7 ± 6%).

## 4. Discussion

FHL2 is an indicator of cell differentiation, from MSCs to osteoblasts. To date, this protein expression is low in HSC cytoskeletal sub-compartments and null in nucleus cells [29]. FHL2 appears here in bone marrow cells of radii obtained from rats after stimulation by high-intensity interval running.

This bone marrow cell response to mechanical stress in vivo could be compared to the response induced in vitro by chemical stimulation (dexamethasone, for instance), for which FHL2 expression induces the differentiation of MSCs in osteoblasts [47].

Our study highlighted the presence of FHL2 in the bone marrow cells in running rats, whereas no FHL2 was observed in sedentary rats. This result indicates that in a situation in which bone is not or is insufficiently exposed to external mechanical stresses, FHL2 is not expressed, whereas FHL2 is synthetized when bone is subjected to sufficient external mechanical stress. Consequently, seven repetitions in blocks of 3 min of running at moderate speed, followed by 2 min of intensive running and 1 min of passive rest, 45 min per day, 5 days per week, for 8 consecutive weeks are deemed to cause sufficient mechanical impact on the locomotor system to induce a FHL2 response in bone marrow cells. Because FHL2 is a protein involved in MSC differentiation in osteoblasts, our results are consistent with the observations from Gonzalo-Encabo et al. (2019) [50], that an exercise involving the repetition of impacts could warrant osteogenic effects on bone status in postmenopausal women.

We hypothesized that FHL2 could be produced endogenously in vivo in bone marrow cells when they are subjected to external mechanical stress, such as when the osteogenic inductor dexamethasone provokes a production of FHL2 in vitro in bone marrow MSCs [38]. By the way of a mechanical stress, FHL2 synthesis in MSCs (as with dexamethasone) could initiate the expression of the transcription factor Runx2, leading to the differentiation of the osteoblast lineage and the mineralization of the extracellular matrix. This could explain the immunostaining observed in cortical bone in the EX group, since differentiation was constantly taking place for osteoblasts close to the endosteal cortical bone. FHL2 could be an indicator for the activation of the mechanisms by which bone marrow MSC differentiate into osteoblasts, before it becomes a transcription factor itself, translocating to the nucleus through its association with the cargo protein IGFBP-5 [51].

FHL2 is a metalloprotein from the zinc finger class. For others such as the Muscle Lim Protein (MLP) in muscular cells, a translocation from the cytoskeleton to the nucleus had been identified [52]. It depends on an activation resulting from a post-transductional response to stress and, for example, to a mechanical stress.

Moreover, Nakawa et al., 2016 [40], reported an FHL2 movement from the cytoskeleton to the nucleus to activate P21 expression. So, even if P21 is involved in the inhibition of the cell proliferation, we cannot exclude that FHL2 could also be involved in another role such as a transcriptional cofactor, or a function mediated by FAK activation, with a translocation to the nucleus.

### How Could FHL2 Be Synthetized in Response to a Mechanical Stimulus?

**Figure 2:**
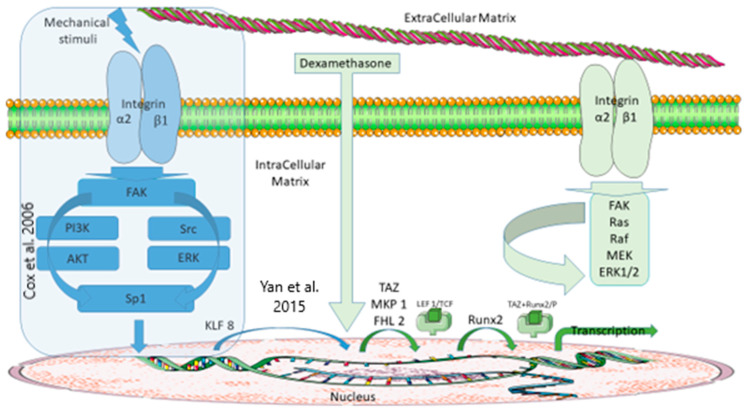
Schematic representation of two signaling pathways involved in FHL2 transcription regulation. **Right-hand side**: Schematic diagram of dexamethasone-induced FHL2 transcription and subsequent activation cascades (adapted from Langenbah and Handschel 2013 [47]). **Left-hand side (frame)**: Signalling pathway leading to the synthesis of FHL2 by mechano-transduction. The FAK would play a key role by activating two transduction signalling pathways that regulate Sp1 (PI3K/AKT, Src/ERK pathways). Sp1 would bind to a KLF8 promoter to initiate KLF8 transcription and KLF8 would initiate the transcription of FHL2 by binding to its promoter.

Among the mechanisms described for cells to achieve extracellular and intracellular medium communication, tensegrity and mechano-transduction allow the translation of the mechanical strains applied to the ECM into an intracellular mechanical stress [53] or an intracellular biochemical message [54]. Numerous transduction signalling pathways have been identified, often involving integrin complexes as the cell gateway. Moreover, integrins have been associated with the differentiation of MSCs into osteoblasts [55]. Consequently, based on a possible response of MSCs to mechanical nano-stimuli involving integrins [56], and with reference to the FHL2-dependent signalling pathways leading to the differentiation of MSCs in osteoblasts, we have tried to understand how bone marrow cells could induce FHL2 synthesis in response to an external mechanical stress (Figure 2).

To answer this question, the literature describes the KLF8 protein as a zinc finger protein with a possible role in transcription [57] and mechano-transduction [27]. Cytoplasm–nuclear localisation [58] is ensured by the presence of an NLS (nuclear localisation signal) fragment [59]. It appears that the synthesis of KLF8 can be initiated at the focal adhesion plate by the activation of FAK [46] and then by the activation of two main signalling pathways, specifically PI3K/AKT [44] and Src/ERK [42], before the transcription factor Sp1 induces its synthesis. It could then be possible to link the expression of KLF8 and FHL2: KLF8 becomes a transcription factor by binding to the gene encoding for FHL2 (binding to the GT box 1 promoter of the GGGTG sequence of nucleic acids -55 and -50) [60,61]. These mechanisms could explain why the lack of KLF8 and the lack of FHL2 have similar effects. For instance, a decrease in KLF8 expression slows down the proliferation of differentiated cells (cancerous osteoblasts) or metastases in osteosarcomas [62], and FHL2 positively affects osteoblastogenesis, bone formation, and bone mass [35]. As displayed in Figure 2, a mechano-transductive pathway could activate the end of the signalling pathway that is known to be activated in vitro by dexamethasone.

In contrast to our results for FHL2, we cannot quantitatively differentiate at the protein level the presence of KLF8 in the BM of trained rats compared with the presence of KLF8 in the BM of untrained rats (14± 4.2% vs. 12.7 ± 6%), and this lack of statistical difference may appear surprising.

In this context, it is necessary to specify that:

KLF8 is expressed at the cellular level for multiple reasons. It is involved in a set of mechanisms to differentiate healthy cells (e.g., for adipocyte production [45]) and during the regulation of cell proliferation (e.g., cancerous tumours [61]).

KLF8 is not exclusively nucleo-plasmatic [43] but also cytosolic out of nuclear adaptative processes.

The immuno-histochemical analyses do not allow to differentiate an overexpression vs. an expression from a quantitative point of view.

It explains firstly the identification of KLF8 in the rat bone marrow cells without mechanical stress induced by running exercise and secondly that we were unable to associate the presence of this protein in a cell sub-compartment as a nuclear adaptative process to exercise.

However, even without being able to conclude an overexpression related to a mechanical stress, the presence of KLF8 in the cell’s nucleus of trained animals is compatible with an activation of the FAK-dependent signalling pathway involving KLF8/FHL2 cooperation.

We emphasize that a subcellular localization and quantification becomes necessary to validate with certainty that running can induce FHL2 transcription by synthesis of nuclear KLF8 (cytoplasmic–nuclear translocation of FHL2 for MSC differentiation remaining a possible and independent mechanism).

The mechanism proposed here could explain (i) why it is possible, when MSCs differentiate in osteoprogenitors, to observe the protein FHL2 in MSCs still engaged in the differentiation phase, and (ii) why, in a healthy differentiated or pre-differentiated breast (cancer) tissue, the cells are devoid of FHL2. Our hypothesis would also explain how, by mechano-transduction, FHL2 could be produced in bone marrow MSCs under mechanical stress and initiate the osteogenic differentiation of the MSCs.

Our hypothesis regarding the mechanism of expression of FHL2 in CSMs is presented in Figure 2, in the framed section. Finally, our hypothesis is similar to that from Tsimbouri et al. (2015) [56], who concluded that bone marrow cells, and in particular MSCs, are able to respond to nano-stimulation and pave the way for adaptive responses.

## Data Availability

The data presented in this study are available on request from the corresponding author.
